# Design of MEMS Microphone Array Integrated System for Pipeline Leakage Detection

**DOI:** 10.3390/mi17010140

**Published:** 2026-01-22

**Authors:** Kaixuan Wang, Yong Yang, Daoguang Liu, Di Song, Xiaoli Zhao

**Affiliations:** 1 School of Information Engineering, Xuzhou College of Industrial Technology, Xuzhou 221140, China; wangkx@mail.xzcit.cn (K.W.);; 2 School of Civil Engineering, Southeast University, Nanjing 211189, China; 3 Special Equipment Safety Supervision Inspection Institute of Jiangsu Province, Nanjing 210036, China; 4 School of Mechanical Engineering, Nanjing University of Science and Technology, Nanjing 210094, China

**Keywords:** micro-electromechanical system (MEMS), microphones array, pipeline, leakage detection, integrated system

## Abstract

Pressure pipelines are widely used in the energy and transportation fields for conveying natural gas, water, etc. Under complex and harsh conditions with long-term operation, this easily leads to leakage, threatening the safe and stable operation of transportation systems. Although acoustic sensors support non-destructive leakage detection, their accuracy is restricted by noise interference and minor leakage uncertainties, and existing systems lack a targeted integration design for pipeline scenarios. To address this, the micro-electromechanical system (MEMS) is specifically designed as an MEMS microphone array integrated system (MEMS-MAIS), which is applied for pipeline leakage detection through data fusion at different levels. First, a dedicated MEMS microphone array system is designed to realize high-sensitivity collection of leakage acoustic data. In addition, the integrated feature extraction and feature-level fusion modules are proposed to retain effective information, and a decision-level fusion module is incorporated to improve the reliability of leakage detection results. To verify the designed system, an experiential platform is established with several microphone data. The results indicate that the proposed MEMS-MAIS exhibits excellent anti-interference performance and leakage detection accuracy of 94.67%. It provides a reliable integrated system solution for pipeline leakage detection and verifying high engineering application value.

## 1. Introduction

As critical infrastructure in energy supply, municipal water conservancy, and petrochemical transportation fields, pressure pipelines undertake the task of conveying high-pressure media such as natural gas, tap water, and crude oil [1]. According to statistics from the International Pipeline and Offshore Contractors Association (IPLOCA), the global length of pressure pipelines has exceeded 3.5 million kilometers, and the scale is still expanding with the development of urbanization and energy interconnection [2]. However, long-term operation under harsh conditions easily leads to micro-cracks and weld defects in pipeline walls, further evolving into leakage accidents [3]. Such leaks not only cause massive waste of resources but also trigger safety hazards of environmental pollution, fire explosions, and road subsidence, seriously threatening the safe and stable operation of urban public systems and industrial production [4].

To address pipeline leakage risks, various non-destructive detection technologies have been developed with different sensors. Specifically, acoustic-based detection methods are widely used due to their advantages of real-time monitoring, low installation cost, and no damage to pipeline structures [5]. Traditional acoustic detection mainly relies on single piezoelectric sensors or ultrasonic probes to collect leakage-induced acoustic signals. However, in actual pipeline scenarios, the acoustic signals generated by minor leaks are extremely weak and easily masked by environmental noise [6]. This leads to low detection accuracy of traditional single-sensor systems, with high false alarm rates exceeding minor leaks [7]. Moreover, existing acoustic detection systems usually use universal acoustic sensors and data acquisition systems, which increase the difficulty and cost of installation and maintenance when applied to ultra-long distance pipeline layouts. Integrated detection systems for pipeline leakage detection with low power consumption, high reliability, and low cost are lacking.

In recent years, micro-electromechanical system (MEMS) technology has provided a new solution for improving acoustic detection performance [8]. MEMS microphones have the advantages of small size, low power consumption, high sensitivity, and strong anti-electromagnetic interference ability [9]. From the perspective of research status, MEMS microphone technology has achieved breakthroughs in both performance optimization and structural innovation [10]. The development of semiconductor microfabrication and flexible packaging processes has reduced production costs while improving detection accuracy in industrial noise environments [11]. In structural research, the focus has shifted from single-microphone devices to array systems for highly reliable performance, and the distributed array layouts and circular array designs tailored to specific scenarios have become research hotspots [12,13]. Additionally, the integration of MEMS microphones with different artificial intelligence algorithms has become a key development direction through built-in noise reduction and feature extraction algorithms. This enhances the ability to process complex acoustic signals.

In terms of MEMS application, MEMS microphones have achieved large-scale deployment across multiple fields, including smartphones, wearables, and smart home devices [14]. The automotive industry uses MEMS microphone arrays for in-car voice interaction and abnormal noise monitoring of engines, leveraging their high stability under extreme temperature fluctuations [15,16]. For pipeline detection, existing applications mainly rely on discrete MEMS sensors for single-point monitoring, lacking integrated array systems designed for the full lifecycle of pipeline operation [17]. This creates a technical gap between mature MEMS technology and practical pipeline leakage detection needs.

Based on this, the MEMS microphone array integrated system (MEMS-MAIS) is designed for pipeline leakage detection, aiming to solve the problems of poor anti-interference ability, low integration, high cost, and low accuracy of existing systems. First, a dedicated MEMS microphone array structure is designed to achieve high-sensitivity collection of leakage acoustic signals. In addition, a feature extraction and feature-level fusion module is integrated into the system to enhance the retention of effective leakage information, and the reliability of leakage judgment is improved by adding a decision-level fusion module. Finally, the hardware and software are integrated for the MEMS-MAIS to ensure its adaptability to pipeline leakage detection under different conditions. To verify the performance of the designed MEMS-MAIS, an experimental platform was built to collect the pipeline leakage acoustic signals under different working conditions. Based on the comparative analysis, the reliability and stability of the proposed MEMS-MAIS are further validated for pipeline leakage detection.

The remaining parts of this paper are organized as follows: Section 2 details the design of the MEMS-MAIS, and the experimental platform is introduced in Section 3. Section 4 presents the experimental results and discussions, and Section 5 summarizes the research.

## 2. Pipeline Leakage Detection Method Through MEMS Microphone Array Integrated System (MEMS-MASI)

### 2.1. Micro-Electromechanical System (MEMS) Microphone Array System Designed for Data Collection

To address the challenges of weak leakage signals and complex environmental interference in pipeline detection, the MEMS microphone array system is designed for acoustic data collection, including an array perception layer, signal conditioning layer, and data transmission layer. The array perception layer, as the front-end signal acquisition unit, consists of multiple MEMS microphones arranged in a specific topology to convert leakage-induced acoustic pressure signals into electrical signals. Additionally, the signal conditioning layer processes weak electrical signals through amplification, filtering, and gain control to suppress noise and ensure signal integrity. Finally, the data transmission layer realizes stable data transmission to the backend computing unit via wireless means, supporting real-time monitoring and subsequent analysis.

Considering the harsh working environment of pipelines and the demand for weak signal detection, the MEMS microphone model selected in this system is the ADI ADMP801 (Analog Devices, Inc. Wilmington, MA, USA). It has a high sensitivity of −35 dBA, which can capture acoustic signals with an amplitude as low as pressure. The power consumption is low with 1 V and 17 µA of current, and the size is small, 3.35 mm × 2.5 mm × 0.98 mm, which is conducive to dense array deployment in limited pipeline installation spaces. In addition, the ADMP801 supports a flat frequency response in the range of 20 Hz–20 kHz, which fully covers the main frequency band of pipeline leakage acoustic signals, avoiding signal distortion caused by frequency response deviation. The parameters of the ADI ADMP801 are shown in Table 1.

The weak electrical signals output by MEMS microphones are amplified and filtered to meet the input requirements of the subsequent analog-to-digital converter. The system selects the TI INA128 (Texas Instruments, Dallas, TX, USA) instrumentation amplifier as the core of the signal conditioning module. It has a programmable gain range from 1 to 10,000, and it can amplify weak leakage signals while suppressing background noise. The parameters of the TI INA128 are shown in Table 2. In addition, a second-order RC low-pass filter is designed in the conditioning circuit to eliminate high-frequency noise above the leakage signal frequency band, further improving the signal-to-noise ratio of the collected data.

**Figure 1:**
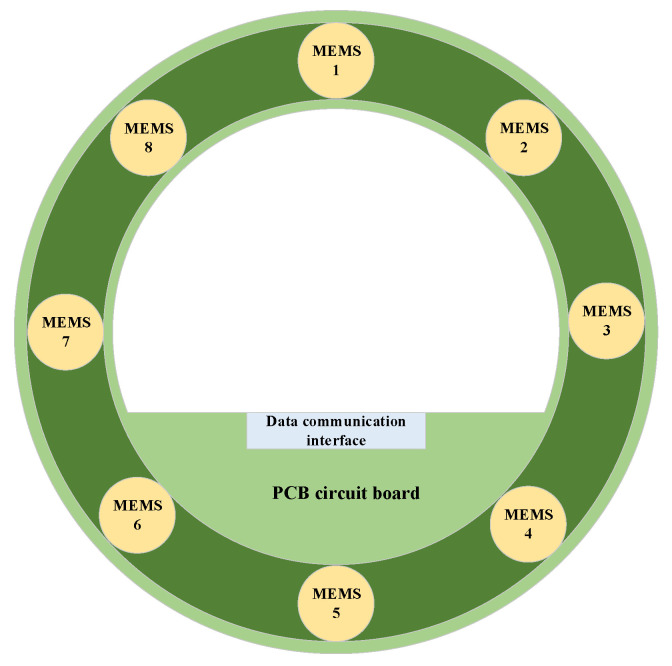
The structure of micro-electromechanical system (MEMS) microphone array topology.

The rationality of the MEMS microphone array topology directly affects the coverage range and accuracy of leakage signal collection. Combined with the cylindrical structure of pipelines and symmetry data collection, a circular–axial combined topology is proposed for the MEMS microphone array, as shown in Figure 1. Specifically, 8 MEMS microphones are evenly arranged along the circumferential direction, and an internal area is created for placing Printed Circuit Board (PCB) circuit boards for data acquisition and control. In addition, the data communication interface is reserved to transmit data to the data transmission layer.

Before the collected signals are transmitted to the backend, a preprocessing module is designed in the system to further optimize data quality, mainly including three functions of automatic gain control, interference filtering, and analog-to-digital conversion (ADC). According to the amplitude of the input signal, the gain of the instrumentation amplifier is dynamically adjusted to ensure that the signal amplitude is always within the input range of 0–3.3 V. This can avoid signal clipping caused by excessive amplitude or loss of weak signals due to insufficient gain. A notch filter with a center frequency of 50 Hz is added to suppress power frequency noise introduced by the system power supply, which is a common interference source in industrial environments. Based on the 16-bit ADC chip of ADS1115 (Analog Devices, Inc. Wilmington, MA, USA), the analog signal is converted into a digital signal with a sampling rate of 44.1 kHz. The sampling rate is 2.2 times the maximum frequency 20 kHz of the MEMS microphone, meeting the Nyquist criterion for the integrity of the digital signal.

Specifically, the number of 8 sensors is only a reference, and more or fewer quantities can be used for the proposed MEMS-MAIS. Additionally, the criteria of a circle radius is determined by the size of the ADI ADMP801 sensor and PCB circuit board, ensuring no sensor overlap for optimal signal reception. It leaves a central area for placing key modules without spatial conflict. The uniform distribution ensures consistent signal reception sensitivity across all directions, avoiding uneven response caused by non-equidistant/random layouts. It combines the symmetry of uniform arrangement and randomness flexibility, avoiding blind spots while simplifying PCB wiring and on-site installation.

### 2.2. Feature Extraction and Feature-Level Fusion

Based on the preprocessed digital signals from the MEMS microphone array system, the robust acoustic features are extracted to characterize pipeline leakage. Considering the time-domain impulse characteristics and frequency-domain energy concentration of pipeline leakage acoustic signals, 6 common and effective acoustic features are selected for feature extraction, including 3 time-domain features and 3 frequency-domain features.

For time-domain features, it includes the peak value (*P*) [18], root mean square (*RMS*) [19], and Kurtosis (*K*) [20]. The peak value is the maximum absolute value of the preprocessed acoustic signal, and it is significantly higher than the background noise due to the leakage signals generating transient pressure pulses. This makes this feature effective for distinguishing leakage from non-leakage states, and the peak value is expressed as

(1) P=max(∣x(n)∣),

where *x*(*n*) is the *n*-th sampling point of the acoustic signal, and *P* is the peak value.

The *RMS* is the square root value for the average of the acoustic signal’s squared values, and it reflects the overall energy of the signal. For the leakage signals, it has a higher square root value than the environmental noise, and it is expressed as

(2) $$RMS=\sqrt{\frac{1}{N}\sum_{n=1}^{N}x{\left(n\right)}^{2}},$$

where *N* is the length of the acoustic signal, and *RMS* is the root mean square.

Kurtosis can measure the impulsivity of an acoustic signal, and the leakage signals are impulsive with a higher Kurtosis value than Gaussian noise. It is expressed as

(3) $$K=\frac{1}{N}\sum_{n=1}^{N}{\left(\frac{x\left(n\right)-\mu }{\sigma }\right)}^{4},$$

where *μ* is the signal mean, *σ* is the standard deviation, and *K* is the Kurtosis value.

In addition, the preprocessed time-domain acoustic signal is converted to the frequency domain via Fast Fourier Transform, and three features are extracted as frequency-domain features. These include the spectral entropy (*SE*) [21], spectral centroid (*SC*) [22], and spectral spread (*SS*) [23]. The *SE* characterizes the uniformity of frequency-domain energy distribution. Leakage signals have energy concentrated in specific sub-bands, and their spectral entropy is lower than that of uniform noise. It is expressed as

(4) $$SE=-\sum_{n=1}^{N}p\left(f_{k}\right)\log_{2}p\left(f_{k}\right),$$

(5) $$p\left(f_{k}\right)=\frac{{\left|X\left(f_{k}\right)\right|}^{2}}{\sum_{n=1}^{N}{\left|X\left(f_{k}\right)\right|}^{2}},$$

where *f_k_* is the *k*-th frequency point, *p*(*f_k_*) is the probability density of *f_k_*, and *SE* is the spectral entropy.

The *SC* is the center of gravity for the frequency spectrum, and it reflects the main energy-concentrated frequency. Specifically, leakage signals have stable spectral centroids, while environmental noise has variable centroids. It is expressed as

(6) $$SC=\frac{\sum_{n=1}^{N}f_{k}\cdot {\left|X\left(f_{k}\right)\right|}^{2}}{\sum_{n=1}^{N}{\left|X\left(f_{k}\right)\right|}^{2}},$$

where *SC* is the spectral centroid.

The *SS* is the quantifiable indicator reflecting the dispersion degree of signal energy in the frequency domain, which has an intrinsic correlation with pipeline leakage acoustic signals due to the physical mechanism of leakage sound generation. It can quantify the deviation of frequency components from the spectral centroid, and it is expressed as

(7) $$SS=\sqrt{\frac{\sum_{k=b1}^{b2}{\left(f_{k}-SC\right)}^{2}\cdot {\left|X\left(f_{k}\right)\right|}^{2}}{\sum_{k=b1}^{b2}{\left|X\left(f_{k}\right)\right|}^{2}}},$$

where *SS* is the spectral spread, and b1 and b2 are the band edges of the leakage-sensitive sub-band.

Combined with the circular array topology of the MEMS microphone system, the all pairwise combination principle is adopted for feature-level fusion. Unlike fixed symmetric pairing, this strategy covers every non-repetitive two-microphone combination. The total of 28 groups of fusion pairs is constructed based on a full-combination design. It can fully leverage the spatial correlation of leakage acoustic signals and avoid information loss caused by limited fixed pairs, as shown in Table 3.

Specifically, when leakage occurs, the acoustic signal energy collected by microphones near the leakage point is significantly higher than that of microphones affected by only background noise. Using energy as the weight ensures that microphones with more effective leakage information contribute more to the fused feature, avoiding the equal weight defect that dilutes valid signals. Therefore, the fusion weight is determined based on the signal energy of a single microphone.

For a fusion pair (microphone *i*, microphone *j*), the weight of each microphone is the ratio of its energy to the total energy of the pair, and it is expressed as

(8) $$\omega_{i}=\frac{E_{i}}{E_{i}+E_{j}},\;\omega_{j}=\frac{E_{j}}{E_{i}+E_{j}},$$

(9) $$E_{i}=RMS_{i}\cdot N,$$

where *E_i_* and *E_j_* are the time-domain energies of microphones *i* and *j*, *ω_i_* and *ω_j_* are the weights of microphone *i* and *j* features, and *ω_i_* + *ω_j_* = 1 to ensure the weight sum is 1.

For each of the 6 extracted features, the values of the two microphones in the pair are fused on the feature-level using their energy weight, and it is expressed as

(10) $$F_{fusion}=\omega_{i}F_{i}+\omega_{j}F_{j},$$

where *F_i_* and *F_j_* are the feature values of microphones *i* and *j*. Each fusion pair outputs a 6-dimensional fused feature vector, and 28 pairs generate 28 sets of fused feature data.

### 2.3. Decision-Level Fusion for Pipeline Leakage Detection

**Figure 2:**
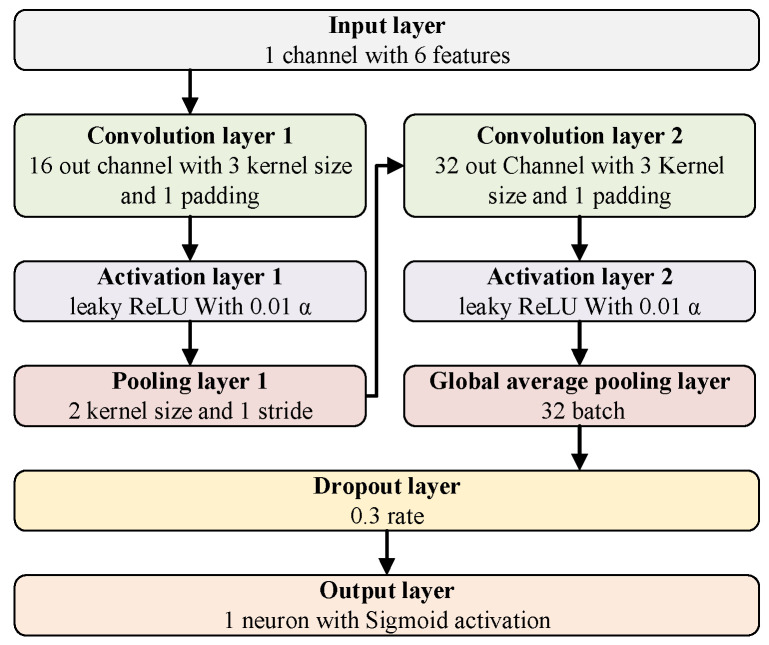
The structure of a lightweight 1D convolutional neural network (1D CNN) for feature classification.

Considering the low dimensionality of fused features and engineering deployment requirements with low computational complexity, a 4-layer lightweight 1D convolutional neural network (1D CNN) is designed as the base classifier. It includes a convolution layer, an activation layer, and a pooling layer, as shown in Figure 2.

The 28 sets of 6-dimensional fused features are first normalized for the range of [0,1] using min-max scaling. Normalization eliminates magnitude differences between features and accelerates model convergence. Each normalized feature vector is then reshaped to match the 1D CNN input format. It is expressed as

(11) $$F_{norm}=\frac{F_{fusion}-F_{\min}}{F_{\max}-F_{\min}},$$

where *F*_max_ and *F*_min_ are the minimum and maximum values of each feature in the training set, and *F_norm_* is the normalized feature.

After training, the 1D CNN model is used to independently predict each of the 28 sets of fused features. For each feature set, the model outputs a prediction probability *p* of [0,1] and a binary result *r* of {0, 1}, where 1 is for leakage, and 0 is for normal. The 28 binary results form a primary decision set *R* = {*r*_1_, *r*_2_,…, *r*_28_}, and the corresponding probabilities form a confidence set *P* = {*p*_1_, *p*_2_,…, *p*_28_}.

The decision-level fusion strategy is improved based on the roulette principle, leveraging the confidence difference between 28 groups of results to suppress the impact of erroneous predictions. The core logic is random elimination and weighted majority voting. The specific steps are as follows:

1. Each prediction result *r_i_* is assigned a weight *ω_ri_*, proportional to its confidence *p_i_*. This reflects the intrinsic correlation between fused features and prediction reliability. The result with higher confidence is more reliable for leakage and normal information, which contributes more to the final decision. It is expressed as
  (12) $$\omega_{ri}=\left\{\begin{array}{l} \frac{p_{i}}{\sum_{j=1}^{28}p_{j}}\;if\;r_{i}=1\;\\ \frac{1-p_{i}}{\sum_{j=1}^{28}\left(1-p_{j}\right)}\;if\;r_{i}=0 \end{array}\right.,$$
  where *r_i_* and *p_i_* are the binary result and prediction probability of the *i*-th output of 1D CNN, and *ω_ri_* is the weight of the prediction result *r_i_*.
2. One group result is randomly removed from the 28 groups. It simulates the roulette spin process and avoids over-reliance on any single result.
3. For the remaining 27 groups, the total weight is calculated for leakage and normal results. If *W_1_* > *W*_0_, the detection result is a leakage condition; otherwise, it is a normal condition. It is expressed as
  (13) $$\begin{array}{l} W_{1}=\sum_{r_{i}=1}\omega_{ri}\\ W_{0}=\sum_{r_{i}=0}\omega_{ri} \end{array},$$
  where *W*_1_ and *W*_0_ are the total weight leakage and normal results.

To avoid randomness errors from a single elimination, the above process is repeated 3 times, and the majority result of the 3 rounds is taken as the final pipeline leakage detection conclusion.

### 2.4. MEMS Microphone Array Integrated System and Its Application

**Figure 3:**
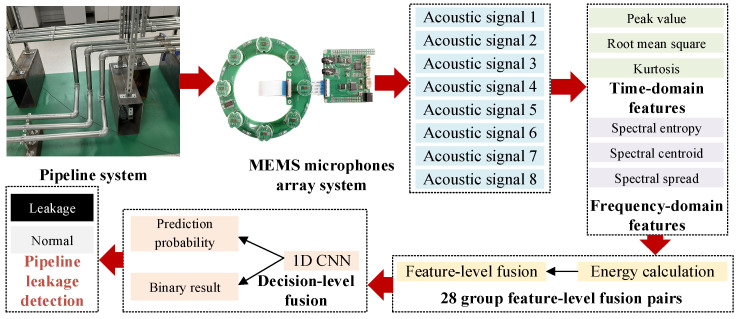
The application of the proposed MEMS microphone array integrated system (MEMS-MAIS).

Based on the designed MEMS microphone array system, feature extraction and feature-level fusion module, and decision-level fusion module, the MEMS-MAIS is proposed for pipeline leakage detection, as shown in Figure 3. The specific steps are as follows:

1. Before formal operation, the MEMS microphone array system is calibrated using a standard sound source with 94 dB SPL and 440 Hz to ensure the sensitivity consistency of all 8 microphones. After deployment, MEMS-MAIS enters the continuous detection mode, starting with signal acquisition and preprocessing.
2. For each preprocessed acoustic signal, the feature extraction module calculates 6-dimensional acoustic features, including time-domain features of P, RMS, and K and frequency-domain features of SE, SC, and SS. Based on the pairwise combination principle, the 8 microphones form 28 non-repetitive pairs. For each pair, the energy weight of each microphone is calculated for feature-level fusion.
3. Based on the proposed lightweight 1D CNN, the 28 sets of fused features are input into the decision-level fusion module for preliminary results and corresponding probabilities. Based on the roulette principle, 1 group of results is randomly eliminated to avoid over-reliance on a single microphone pair, and the total weights of the remaining 27 groups are summed for pipeline leakage detection.

## 3. Experimental Platform Establishment

**Figure 4:**
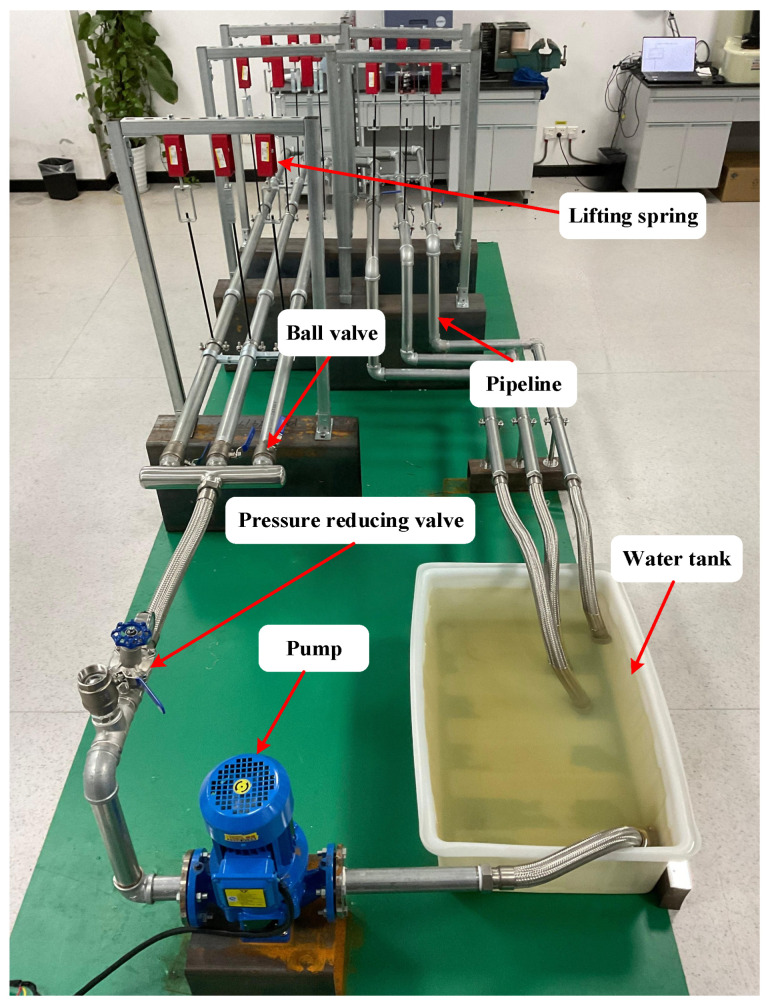
The water pipeline experimental platform.

To further evaluate the effectiveness of the proposed MEMS-MAIS, the water pipeline experimental platform is established with several devices under different conditions. It can simulate the acoustic characteristics of urban water supply pipelines and oil transmission pipelines, as shown in Figure 4.

The platform consists of five core modules, including the power drive module, fluid circulation module, pressure regulation module, pipeline module, and leakage simulation module. The detailed parameters of the experimental devices are shown in Table 4.

The power drive module consists of a 0.75 kW three-phase asynchronous motor and a variable frequency drive, which is used to adjust the motor speed to stabilize the water pump’s output flow at 5.6 m^3^/h, matching the actual operating conditions of small-to-medium diameter water supply pipelines. The fluid circulation module comprises a 100 L water tank and a filter installed at the tank outlet to remove impurities. The pressure regulation module is equipped with a precision pressure-reducing valve and a pressure gauge. The pressure is adjusted by rotating the valve core, and the system is allowed to stabilize for 5 min after each adjustment to ensure pressure uniformity. The pipeline module uses DN32 galvanized steel pipes, which are connected by flange joints. The pipeline is fixed on a lifting spring system to simulate the three-degree-of-freedom micro-vibration of industrial pipelines. The leakage simulation module has a ball valve with an adjustable opening degree as the leakage source, and a typical pipeline system is designed with three pipelines at three degrees of freedom vibration.

**Figure 5:**
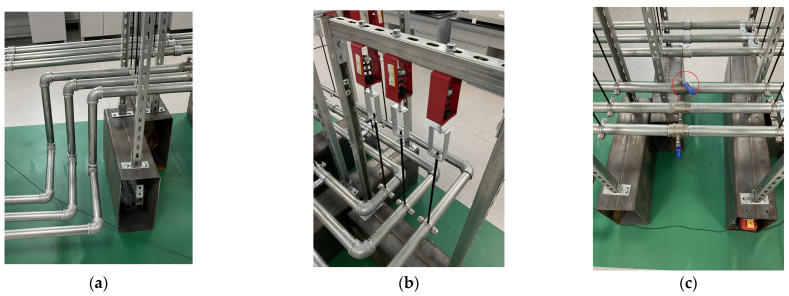
The special structure of experimental platform: (**a**) typical pipeline system; (**b**) lifting spring system; (**c**) half-open ball valve.

In addition, the ball valve is utilized to simulate the leakage condition of the pipeline. Specifically, when the ball valve is closed, there is no leakage of water in the pipeline, but when the ball valve is opened, the pipeline leaks and water flows out of the pipeline back to the water tank. It can simulate the leakage situation during the actual operation of the pipeline by setting up a ball valve to half-open and close, as shown in Figure 5.

All experiments in this study are carried out on a self-built physical water pipeline platform, and no simulation models or simulation software are used. The acoustic data of pipeline leakage and normal conditions are directly collected from the physical system to ensure the authenticity and reliability of the experimental results.

## 4. Results and Discussion

### 4.1. Results

**Figure 6:**
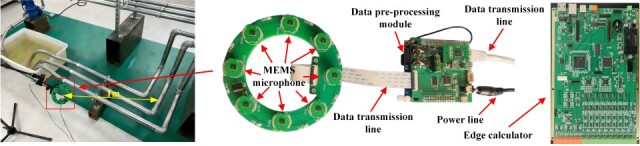
The MEMS microphone array integrated system.

Based on the MEMS microphone array system and serval signal process module of feature extraction, feature-level fusion, and decision-level fusion, the MEMS-MAIS is obtained with eight MEMS microphones, a data preprocessing module, a data transmission module, and an edge calculator. The MEMS microphone array integrated system and its location on the pipeline experimental platform are shown in Figure 6.

The MEMS microphone array is installed on the side of a typical pipeline system through a sensor bracket with about 1 m distance. Additionally, the collected data are communicated through shielded flat wires, and the edge calculator synchronously stores raw data and processed features in a 16 GB microSD card for post-experiment verification. Each experimental condition is repeated five times independently by two different researchers. The relative standard deviation of detection accuracy across repetitions is below 1.2%, confirming the stability of the experimental setup and data.

The eight MEMS microphones is arranged with the circular–axial combined topology to collect acoustic signals, which is transmitted to the data preprocessing module for preprocessing adjustment. Specifically, all MEMS microphones are calibrated to ensure their consistency and accuracy. If the sensitivity deviation between any two microphones exceeds ±1 dBA, we adjust the bias voltage of the corresponding microphone to ensure consistency. The processed data are transmitted to the edge calculator for feature extraction, feature-level fusion, and decision-level fusion.

Although the microphone pairs are so close together in the array (a few centimeters) and the array is 1 m away from the source, there are significant differences in the parameters of each microphone within a pair, including amplitude difference and phase difference. Leakage acoustic signals propagate outward in the form of spherical waves rather than plane waves. The amplitude of spherical waves is inversely proportional to the propagation distance. The amplitude difference between the two microphones in a pair is calculated as

(14) $$\Delta A=\left|\frac{1}{r}-\frac{1}{r+d}\right|\approx \frac{d}{r^{2}},$$

where Δ*A* is the amplitude difference, and *r* and *d* are the source distance and spacing distance.

For the designed MEMS microphone array, the diameter of the array is 4 cm, and the source distance and spacing distance are 1 m and 4 cm, respectively. Based on *d* = 4 cm and *r* = 1 m, the amplitude difference *ΔA* is about 4%, which is sufficient to be captured by the high-sensitivity ADMP801 microphones and is amplified during feature extraction. Additionally, the phase difference is determined by the path difference, and it is expressed as

(15) $$\Delta \varphi =\frac{2\pi d}{\lambda },$$

where Δ*φ* is the phase difference, and *λ* is the acoustic wavelength.

Generally, pipeline leakage generates high-frequency ultrasonic “whistles” with a high frequency range from 20 kHz to 100 kHz, due to high-speed fluid jetting through small leak holes. However, this high-frequency ultrasonic energy will attenuate rapidly in air and pipeline walls. For typical leakage signal frequencies from 1 kHz to 2 kHz, the acoustic wavelength *λ* is 0.17–0.34 m. The phase difference ranges from 0.23π to 0.47π rad. This phase difference introduces distinct time-domain waveform characteristics for each microphone, forming the basis of spatial feature discrimination.

To test the performance of the designed MEMS-MAIS, the acoustic data are collected from the water pipeline experimental platform. Specifically, the sampling frequency is 44,100 Hz with 1 s, and the pipeline is operated at three pressures of 5 PSI, 10 PSI, and 15 PSI, which can simulate different working conditions. Through the ball valve, two conditions of the pipeline are obtained to simulate the normal and leakage conditions. A total of 100 sets of acoustic data are collected for each operating condition, and there are 2 × 3 × 8 × 100 (2 pipeline conditions, 3 pressure conditions, 8 MEMS microphones, and 100 sets of data) acoustic data obtained to establish the pipeline leakage dataset under different conditions, as shown in Table 5.

Therefore, the designed MEMS-MAIS is applied for the pipeline leakage dataset. First, six common and effective acoustic features are extracted from the time and frequency domain, including peak value, root mean square, Kurtosis, spectral entropy, spectral centroid, and spectral spread. According to the proposed feature-level fusion rule, 28 groups of fusion pairs are constructed for eight MEMS microphones, and the extracted features obtained from different MEMS microphones are fused on the feature level by calculating their signal energy. Finally, there are 28 × 2 × 100 acoustic samples with six fused features for one condition.

Specifically, the 3σ rule is employed to remove outlier samples where any of the six extracted features deviate by more than three standard deviations from the mean value of each feature. After normalization for the range of [0,1], the training and testing dataset is established based on the division ratio of 7 and 3. For one condition, there are 2 × 2 × 70 and 28 × 2 × 30 acoustic samples for the proposed lightweight 1D CNN training and testing. The training and testing acoustic samples are shown in Table 6.

**Figure 7:**
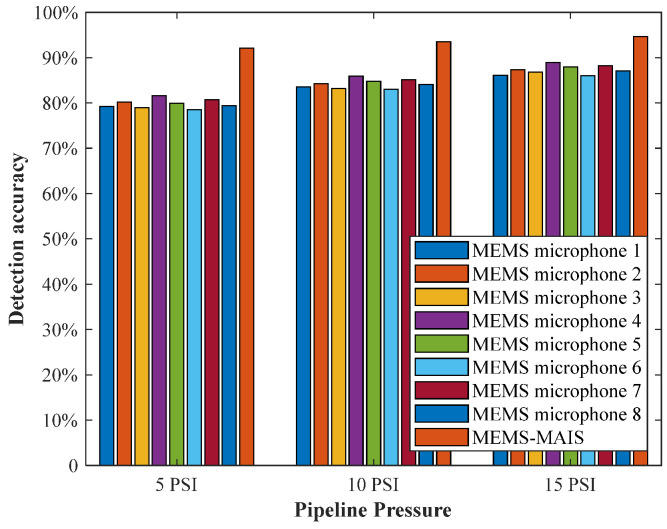
The result of pipeline leakage detection.

Based on the trained 1D CNN model, the prediction probability and binary result are obtained for 28 × 2 × 30 acoustic samples. Finally, the decision-level fusion result is obtained through random elimination and weighted majority voting. Based on the five-fold cross-validation strategy, the leakage of the pipeline is detected under different pressure and environmental conditions. The accuracy is compared with eight raw MEMS microphone data, as shown in Figure 7.

Clearly, the results of pipeline leakage detection are relatively stable under different pressures, and they reach poor performance for single MEMS microphone data. By fusing raw acoustic data on the feature and decision level, the detection accuracy is further improved, and the highest accuracy reaches 94.67% at a pressure of 15 PSI condition. The reason is that the leakage information generated is more obvious when the pipeline operates under excessive pressure, which is more conducive to the accurate detection of pipeline leakage by the proposed MEMS-MAIS. Overall, the MEMS-MAIS maintains a narrow accuracy fluctuation range across 5 PSI–15 PSI, confirming its ability to adapt to varying pipeline pressure conditions and reliably detect leaks in the pipeline.

### 4.2. Discussion

To validate the contribution of each core component in the MEMS-MAIS, an ablation experiment is designed by comparing three types of data under the three pressure conditions, including the single MEMS microphone condition, feature-level fusion condition, and decision-level fusion condition.

Groups A and B are the average detection accuracy of eight individual MEMS microphones using raw time and frequency domain signals without feature extraction. By extracting the six significant features in two domains, the average accuracy is calculated for Group C. Based on the 28 groups of MEMS microphone feature-level fusion pair, the average accuracy is obtained for Group D. In addition, the results of the MEMS-MAIS detection are employed for Group E, which is the final result of the designed system. All experiments are repeated five times under each pressure condition to reduce random errors, and the average accuracy is shown in Table 7.

The results show a clear upward trend in detection accuracy from Group A to Group E, reflecting the incremental value of each technical improvement. Specifically, Group A, Group B, and Group C obtain similar results, indicating that the selected six features can represent the original data well. This reflects the importance of feature extraction and data dimensionality reduction. Furthermore, Group D reaches the average accuracy of 87.94%, which outperforms the above three results. This highlights the advantage of multi-microphone feature fusion in compensating for single-microphone spatial limitations. Finally, the proposed MEMS-MAIS achieves the highest accuracy, validating the effectiveness of the full system design.

**Figure 8:**
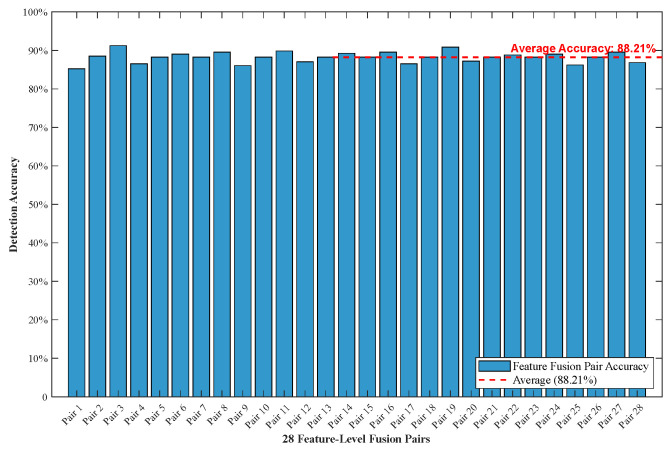
The detection result of 28 feature-level fusion pairs.

To illustrate the performance of the feature-level fusion, the accuracy of 28 feature-level fusion pairs is calculated based on the trained 1D CNN model. The detection accuracy of each pair is obtained through three repeated experiments to reduce random errors. Taking the pressure of 10 PSI as an example, the detection accuracy is shown in Figure 8.

Clearly, the 28 feature fusion pairs exhibit stable detection performance under 10 PSI with the average accuracy of 88.21%, and it has a narrow accuracy fluctuation range from 85.21% to 91.81%. In contrast to the relatively poor performance of single MEMS microphone data, the feature-level fusion module effectively integrates complementary acoustic information, significantly improving detection reliability. This confirms that the proposed feature-level fusion strategy can effectively use the significant information and enable the MEMS-MAIS to adapt to feature extraction and fusion in practical pipeline operations and reliably detect leakage.

**Figure 9:**
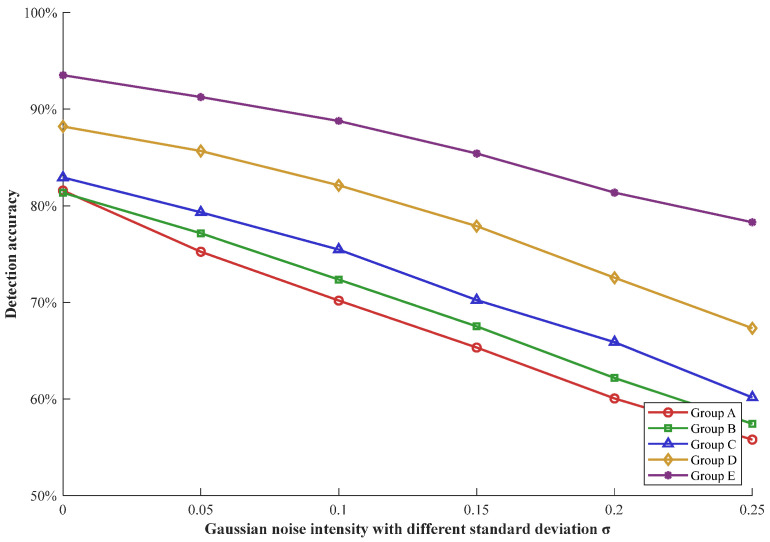
Anti-noise performance of different groups under different Gaussian noise.

To evaluate the stability and anti-noise performance of the proposed MEMS-MAIS, different intensities of Gaussian noise are added to the raw MEMS microphone acoustic data. The detection accuracy of the above five experimental groups is tested under each noise intensity, including Group A, Group B, Group C, Group D, and Group E. Taking a pressure of 10 PSI as an example, the anti-noise performance of each group is shown in Figure 9.

Clearly, the detection accuracy of all groups shows a downward trend with the increase in Gaussian noise intensity, but the decline rate and residual accuracy vary significantly among groups. Group A and Group B exhibit the poorest anti-noise performance, which is because raw acoustic signals are highly susceptible to Gaussian noise interference and lack discriminative features to distinguish leakage signals from noise. Group B performs slightly better than Group A but still shows a sharp accuracy decline, confirming that single-microphone raw signals have limited anti-noise capability. By extracting six significant features, the anti-noise performance of Group C is improved, based on a slight decrease in accuracy. Furthermore, Group D maintains higher accuracy than Group C, as multi-source feature fusion aggregates complementary information to offset noise interference. The proposed MEMS-MAIS of Group E demonstrates the strongest anti-noise stability with little decrease in detection accuracy. The reason is that the MEMS-MAIS integrates feature-level fusion with hybrid decision-level fusion, which can further suppress noise and enhance the robustness of leakage detection.

To simulate authentic working conditions, a professional sound level meter is employed to collect industrial background noise from the water supply pipeline station, which is the typical pipeline operation site. The noise primarily includes equipment operation sounds, air flow noise, and ambient environmental noise. It is played during the data collection process to obtain the industrial noisy pipeline leakage dataset. Based on the proposed system and method, the noiseless and noisy data are processed using the same model and parameters. The compared results are shown in Table 8.

Even under complex real-world noise conditions, the proposed MEMS-MAIS maintains a high detection average accuracy of 90.19% for three pressure conditions. Compared with the noiseless scenario, the average accuracy decreases by only about 2%, indicating strong robustness against practical interference. The accuracy decline is mild when introducing real-world noise. This result demonstrates the effectiveness and reliability of noise resistance. Overall, the designed MEMS-MAIS maintains a narrow accuracy decline range under increasing Gaussian noise, confirming its strong anti-noise performance and stability for practical pipeline leakage detection.

To quantify the computational complexity for edge computing deployment, two core indicators are employed, including parameter count (Params) [24] and floating-point operations per second (*FLOPs*) [25], which are widely used in edge AI deployment research. The parameter count is calculated by summing the trainable parameters of each layer of the 1D CNN. For the global average pooling layer and dropout layer, zero trainable parameters are applied for parameter count. The results are shown in Table 9.

*FLOPs* are calculated based on the convolution operation formula, and it is expressed as

(16) FLOPs=2×Cin×K×Hout×Cout

where *Cin* is the input channel, *K* is the kernel size, *Hout* is output feature map length, and *Cout* is output channel.

The input feature length is six-dimensional fused features, and the *FLOPs* of Convolution layer 1 and Convolution layer 2 are 2 × 1 × 3 × 6 × 16 = 576 and 2 × 16 × 3 × 6 × 32 = 18,432. For the Output layer, the *FLOPs* are 2 × 32 × 1 = 64 for fully connected multiplication and addition. Total *FLOPs* per inference are 576 + 18,432 + 64 = 19,072 (≈19.1k *FLOPs*), which belongs to the ultra-lightweight model category suitable for edge deployment.

Specifically, the selected ADI ADMP801 MEMS microphone offers tailored advantages for harsh pipeline scenarios, including an ultra-small size, enabling dense circular–axial array deployment and multi-point layout along ultra-long pipelines, high sensitivity for capturing weak leakage signals, low power consumption supporting long-term continuous operation, and robust environmental adaptability, matching industrial pipeline conditions. The proposed MEMS-MAIS is designed for on-line and real-time monitoring, leveraging the lightweight 1D CNN and edge computing. It outputs binary leakage/normal results and triggers local LED alerts for monitoring platform u, which meets real-time emergency response requirements. With a measurement range of 0–15 PSI, it is consistent with small-to-medium diameter municipal and petrochemical pipelines, and the system differs significantly from existing technologies through its pipeline-specific circular–axial topology and fully integrated hardware design, energy-weighted feature-level fusion and roulette-based decision-level fusion, and edge-deployable lightweight model, collectively achieving a high detection accuracy of 94.67% and strong reliability under complex noise conditions.

To analyze the influence of the number of sensors and pairs, three sensor quantities and five compared pairs are tested under the same experimental conditions. When a smaller number of sensors/pairs has been selected, the classifier has been retrained, and the results are shown in Table 10 and Table 11. Specifically, the accuracy increases sharply with sensor count, but the marginal gain diminishes. More sensors will result in higher costs, sizes, and energy consumption. The number of eight sensors is only a reference, and more or fewer quantities can be used for the proposed MEMS-MAIS. In addition, the accuracy degrades approximately linearly as the number of pairs decreases below 20. When pairs are below 15, degradation accelerates because the system loses critical spatial correlation information.

## 5. Conclusions

To detect the leakage of pipelines, the MEMS-MAIS is designed based on eight MEMS microphones and an information fusion strategy. Specifically, the MEMS microphone array system is designed to effectively collect acoustic data, which are preprocessed and transmitted for feature extraction. Based on six significant time and frequency features, the feature-level fusion module is presented to obtain 28 non-repetitive feature-level fusion pairs. The preliminary results and corresponding probabilities are obtained based on the proposed lightweight 1D CNN, and the final detection results are obtained through the proposed decision-level fusion module. The experiential results and comparative analysis indicate that the proposed MEMS-MAIS can reach a high leakage detection accuracy of 94.67%. By testing under different pressure and noise conditions, the reliability and stability are further verified for the proposed MEMS-MAIS on pipeline leakage detection. This study provides a feasible technical solution for low-cost, high-reliability pipeline leakage detection based on MEMS microphones.

Future research will focus on expanding the noise types to include industrial background noise and mechanical vibration noise, which can further verify its anti-interference ability. In addition, the 1D CNN model and fusion strategy can be optimized to reduce the computational complexity of the MEMS-MAIS, enabling its deployment on edge computing devices for real-time pipeline leakage monitoring. Another critical direction is to incorporate beamforming techniques to fully leverage the spatial information of all sensors, and future experiments will be carried out to validate whether this configuration can achieve higher detection performance, especially for weak leakage signals in complex noise environments.

## Figures and Tables

**Table 1:** The parameters of the ADI ADMP801.

Parameters	Value
Size	3.35 mm × 2.5 mm × 0.98 mm
Equivalent input noise	27 dBA SPL
Frequency response	20 Hz–20 kHz
Power consumption	1 V and 17 µA of current
Start-up time	0.8 s startup to within 0.2 dB of 1 kHz
Sensitivity	35 dBA
Storage temperature range	−55 °C to +150 °C
Sound pressure level	160 dB

**Table 2:** The parameters of the TI INA128.

Parameters	Value
Voltage gain	1–10,000
Noise at 1 kHz	8 nV√Hz
Common mode rejection ratio	120 dB
Bandwidth at min gain	1.3 MHz
Operating temperature range	−40 °C to +120 °C

**Table 3:** A total of 28 groups of MEMS microphone feature-level fusion pairs.

No.	Sensor Pair	No.	Sensor Pair	No.	Sensor Pair	No.	Sensor Pair
1	(1, 2)	8	(2, 3)	15	(3, 5)	22	(4, 8)
2	(1, 3)	9	(2, 4)	16	(3, 6)	23	(5, 6)
3	(1, 4)	10	(2, 5)	17	(3, 7)	24	(5, 7)
4	(1, 5)	11	(2, 6)	18	(3, 8)	25	(5, 8)
5	(1, 6)	12	(2, 7)	19	(4, 5)	26	(6, 7)
6	(1, 7)	13	(2, 8)	20	(4, 6)	27	(6, 8)
7	(1, 8)	14	(3, 4)	21	(4, 7)	28	(7, 8)

**Table 4:** The detailed parameters of the experimental devices.

Devices	Parameters
Water pump	16 m lift, 5.6 m^3^/h flow rate, 2900 rpm rotation speed
Motor	Three-phase asynchronous motor, 0.75 kW, 380 V
Water tank	100 L with 605 mm × 935 mm × 250 mm
Pressure reducing valve	0–220 PSI pressure range
Pipeline	DN 32, galvanized steel pipe

**Table 5:** The pipeline leakage dataset.

Pressures	Pipeline Conditions	MEMS Microphones	Signal Length
5 PSI	Normal	Sensor 1	100 × 44,100
		Sensor 2	100 × 44,100
10 PSI		Sensor 3	100 × 44,100
		Sensor 4	100 × 44,100
	Leakage	Sensor 5	100 × 44,100
15 PSI		Sensor 7	100 × 44,100
		Sensor 8	100 × 44,100
		Sensor 9	100 × 44,100

**Table 6:** The training and testing dataset.

Pressures	Label	Pipeline Condition	Samples	Training Data	Testing Data
5 PSI	0	Normal	28 × 100	28 × 2 × 70	28 × 2 × 30
	1	Leakage	28 × 100		
10 PSI	0	Normal	28 × 100	28 × 2 × 70	28 × 2 × 30
	1	Leakage	28 × 100		
15 PSI	0	Normal	28 × 100	28 × 2 × 70	28 × 2 × 30
	1	Leakage	28 × 100		

**Table 7:** The result of the ablation experiment.

Experimental Group	Detection Accuracy at Different Pressures			Average Accuracy
	5 PSI	10 PSI	15 PSI	
Group A	80.76%	81.57%	80.15%	80.83%
Group B	82.12%	81.34%	81.05%	81.50%
Group C	80.43%	82.92%	80.41%	81.25%
Group D	86.45%	88.21%	89.16%	87.94%
Group E	92.08%	93.51%	94.67%	93.42%

**Table 8:** The results of different noise conditions.

Different Noise Conditions	Detection Accuracy at Different Pressures			Average Accuracy
	5 PSI	10 PSI	15 PSI	
Noiseless	92.08%	93.51%	94.67%	93.42%
Noisy	88.57%	91.68%	90.33%	90.19%

**Table 9:** The result of the parameter count.

Layers	Details			Params
	Channels	Kernel Size	Padding	
Convolution layer 1	16	3	1	1 × 3 × 16 + 16 = 64
Convolution layer 2	32	3	1	16 × 3 × 32 + 32 = 1568
Output layer	-	32	-	32 × 1 + 1 = 33
Total value	64 + 1568 + 33 = 1665 (≈1.67 k parameters)			

**Table 10:** The comparison of results of different sensor numbers.

Number of Sensors	Detection Accuracy at Different Pressures			Average Accuracy
	5 PSI	10 PSI	15 PSI	
4	81.93%	82.40%	82.78%	82.37%
6	88.21%	89.22%	88.70%	88.71%
8	92.08%	93.51%	94.67%	93.42%

**Table 11:** The comparison of results of different pairs.

Number of Pairs	Detection Accuracy at Different Pressures			Average Accuracy	Accuracy Degradation
	5 PSI	10 PSI	15 PSI		
28	92.08%	93.42%	94.67%	93.42%	-
27	91.78%	93.12%	94.46%	93.12%	0.30%
20	90.12%	91.50%	92.64%	91.42%	2.00%
15	86.70%	89.31%	88.05%	88.02%	5.40%
10	82.10%	83.70%	85.06%	83.62%	9.80%
5	74.50%	78.06%	76.40%	76.32%	17.10%
1	65.16%	60.20%	62.80%	62.72%	30.70%

## Data Availability

Data will be made available upon request.
